# In vitro and in vivo antimalarial activity and chemical profiling of sugarcane leaves

**DOI:** 10.1038/s41598-022-14391-8

**Published:** 2022-06-17

**Authors:** Jude E. Okokon, Rebecca Mobley, Utibe A. Edem, Augustine I. Bassey, Idowu Fadayomi, Falko Drijfhout, Paul Horrocks, Wen-Wu Li

**Affiliations:** 1 Department of Pharmacology and Toxicology, Faculty of Pharmacy, University of Uyo, Uyo, Nigeria; 2 School of Medicine, Keele University, Staffordshire, ST5 5BG UK; 3 Department of Clinical Pharmacology and Therapeutics, Faculty of Basic Clinical Sciences, University of Uyo, Uyo, Nigeria; 4 School of Pharmacy and Bioengineering, Keele University, Stoke-on-Trent, ST4 7QB UK; 5 School of Chemical and Physical Sciences, Keele University, Staffordshire, ST5 5BG UK

**Keywords:** Analytical chemistry, Drug development, Preclinical research, Antimicrobials, Parasitology, Drug discovery, Microbiology

## Abstract

*Saccharum officinarum* Linn. (sugarcane, Family-Poaceae) is employed in Ibibio traditional medicine for the treatment of various infections and diseases such as malaria. We This study aims to assess the antiplasmodial effect of the leaf extract and fractions on human malaria parasite (*Plasmodium falciparum*) in vitro, and rodent malaria parasite (*P. berghei*) in vivo, and analyse the bioactive components of the active fraction(s). The leaf extract and fractions of *S. officinarum* were prepared and their growth inhibitory effects tested against the chloroquine resistant *P. falciparum* strain (Dd2) and *P. berghei* infection in mice. An acute toxicity of the extract was determined. A combination of gas chromatography and liquid chromatography-mass spectrometry, and nuclear magnetic resonance spectroscopy was applied for metabolites profiling of crude extract and active fractions. The leaf extract and fractions demonstrated moderate activity against *P. falciparum* with the dichloromethane fraction producing the most potent activity (EC_50_ = 15.4 µg/mL). The leaf extract (170–510 mg/kg, p.o., LD_50_ = 1732 mg/kg) and fractions demonstrated significant (p < 0.05–0.001) effect on *P. berghei* infection in prophylactic  tests as well as in established infection with *n*-butanol fractions producing the highest effect. An unusual sulphur-containing compound, dilaurylthiodipropionate, fatty acids, phenolic acids, flavonoid and flavonoid glycoside were identified in the active fractions. These results give credence to the use of sugarcane leaves as malarial remedy locally by confirming the in vitro and in vivo antiplasmodial potential of leaf extract/fractions of *S. officinarum*.

## Introduction

Malaria is endemic across tropical and sub-tropical regions of the world with an estimate of 241 million malaria cases and 627,000 deaths in 2020^1^. The increase in these numbers over 2019 represent both a halt in the previous decline in morbidity and mortality as well as the impact of the COVID-19 pandemic. About 95% of malaria deaths across the world are in 31 countries, with Nigeria particularly affected.

Plants provide a valuable reservoir for antimalarial drug discovery^2–5^, with the front-line drug artemisinin representing a particular example of the efficacy of this source. Previously we have evaluated a number of medicinal plants and/or their components^6–8^, semi-synthetic^8,9^ and biotransformed analogues^10^ in vitro and in vivo for the development of potential treatment of malaria. *Saccharum officinarum* Linn. (Family-Poaceae), commonly known as sugarcane, is found across tropical and subtropical areas of the world. It is the world's largest crop with about 1255 million tons per year of cane or 55 million tons per year of sucrose and is estimated to be cultivated in more than 90 countries^11^. Sugarcane is also employed in the management and treatment of various diseases and infections in traditional medicines^12,13^. SAABMAL, an ethnomedicinal polyherbal formulation containing *S. officinarum,* is used in Nigeria to treat uncomplicated malaria infection^14^. The reported biological activities of the leaf extracts also include anti-hyperglycaemic, anti-hyperlipidaemic, antioxidant^15,16^, antidepressant and anticonvulsant^17^, as well as analgesic^18^ activities. Phytochemical investigation of sugarcane juice, and other plant parts, disclosed the presence of long-chain alcohols and acids, glycosides, phytosterols, saponins, tannins, and flavonoids^13,19,20^. Coutinho et al.^20^ identified a variety of flavones and simple phenolics as well as their derivatives in the leaves of *S. officinarum*. However, to date, there remains to be an evaluation of *S. officinarum* antimalarial properties. Here, we investigate in vitro and in vivo antiplasmodial activities of *S. officinarum* leaf extract and fractions as well as characterization of the phytochemical constituents*.*

## Materials and methods

### Plant materials

Fresh leaves of sugarcane were collected from Medicinal Plants farm of Faculty of Pharmacy, University of Uyo, Akwa Ibom State in June, 2020 after acquiring permission and approval from Faculty of Pharmacy, University of Uyo. The leaves were further identified and authenticated as *Saccharum officinarum* Linn. by Prof. Margaret Bassey in the Department of Botany and Ecological studies, University of Uyo, Uyo, Nigeria and a voucher specimen (UUPH 215b) was deposited at the herbarium of the Department of Pharmacognosy and Natural Medicine of the University. All the procedures and collection of plant material was done in accordance with local and national guidelines and regulations.

### Extraction and fractionation of the plant materials

Fresh leaves of *S. officinarum* were washed, chopped into smaller pieces and dried at room temperature for two weeks. The leaves were further reduced to powder using electric grinder. The leaf powder (2 kg) was soaked in 50% ethanol (7.5 L) at room temperature for 3 days. It was thereafter filtered, and the liquid filtrate was concentrated to dryness in *vacuo* under 40 °C using a rotary evaporator (BuchiLab, Switzerland). The crude extract (50.0 g) was dissolved in water (200 mL) and partitioned using *n*-hexane, dichloromethane (DCM), ethyl acetate and *n*-butanol (4 × 500 mL each) to obtain the solvent and aqueous fractions. The calculated yields of the extract and fractions were crude extract-3.52%, *n*-hexane-0.12%, DCM-0.16%, ethyl acetate-0.14%, and *n*-butanol-0.84%. The extract and fractions were stored at  4 °C in a refrigerator.

### In vitro growth inhibition assays

The parasite strain used for in vitro testing was the Dd2^luc^ chloroquine-resistant strain of *P. falciparum* (obtained from Liverpool school of Tropical Medicine and Hygiene, Liverpool, UK)^21^. Parasites were maintained as a continuous culture at 2% haematocrit (HCT), 1–5% parasitaemia and incubated at 37 °C^22^. Supplemented RPMI-1640 medium was changed daily^21^. Extracts or fractions were added to 96-well microplates, with a starting concentration of 700 µg/mL, and two-fold serial dilution was carried across the plate. *P. falciparum* culture was added to all wells, with a final 1–2% parasitaemia of trophozoites (18–28 h post-infection) at a 2% HCT. A positive control of untreated infected RBCs was used to represent 100% growth of parasites. A negative control of infected RBCs treated with a supralethal dose of chloroquine (10 µM) represented 0% growth. Plates were incubated at 37 °C for 48 h in an incubator supplied with 1% oxygen, 3% carbon dioxide and 96% nitrogen.

Malaria SYBR Green I fluorescence (MSF) assays^23^ of parasite growth were carried out for the preliminary four-point screen. SYBR Green I dye (Thermo Scientific, UK) was diluted in 1× MSF Lysis buffer (20 mM Tris HCl, pH 7.5, 5 mM EDTA, 0.08% w/v saponin and 0.08% w/v Triton X-100) at a 1:5000 ratio. To a black 96-well plate, 100 µL of culture from the incubated plate was transferred followed by 100 µL of 1× MSF lysis buffer and dye. This plate was left to incubate for one hour in the dark. The fluorescence signal was then read using Glomax Multi Detection System (Promega, UK) using the blue fluorescence module (excitation 490 nm: emission 510–570 nm). Parasite growth on the nine-point growth inhibition curves, used to estimate EC_50_^,^  were measured using a luciferase bioluminescence assay^21^. To a white 96-well plate, 40 µL of culture from the test plate, 10 µL of 5× passive lysis buffer (Promega, UK) and 50 µL of luciferase substrate (Promega, UK) were added. The bioluminescent signal was recorded using the Glomax Multi Detection. EC_50_ values were estimated from curves plotted of normalized parasite growth versus log10-transformed concentration of extract/fractions (GraphPad v5.0, Prism).

### Experimental animals

Swiss albino mice (18–25 g), male and female, used in the study were obtained from the University of Uyo animal facility. They were housed in standard plastic cages in a well-ventilated room (28.0 ± 2 °C; 12 h day/light cycle). The mice were placed on a standard pelleted diet and water ad libitum. The protocols of animal experiments were carried out in accordance with the National Institute of Health Guide for the Care and Use of laboratory Animals^24^. The study was approved by University of Uyo’s Animal Ethics Committee (UU/CHS/AE/21/068).

### Drug administration

All treatments: extract, fractions, chloroquine and pyrimethamine, in this study were administered orally using a stainless metallic feeding cannula.

### Acute toxicity testing

Acute toxicity testing was carried out by determining the median lethal dose (LD_50_) of the extract intraperitoneally using a modified method of Lorke^25^. Different groups of mice (n = 3) were administered with varying doses of the extract (100–5000 mg/kg). Physical signs of toxicity as well as mortality were recorded in each group within 24 h. The LD_50_ value was calculated as geometrical means of the minimum dose producing 100% mortality and the maximum dose producing 0% mortality according to the method of Lorke^25^.

### *Plasmodium berghei* inoculation

The ANKA strain of *P. berghei* was provided from the National Institute of Medical Research (NIMER), Yaba Lagos, Nigeria. Each mouse used in the experiment was inoculated intraperitoneally with 0.2 mL of infected blood containing about 1 × 10^7^* P. berghei* parasitized erythrocytes collected from an infected mouse with 20–30% parasitaemia. The inoculum consisted of 5 × 10^7^ *P. berghei* infected erythrocytes per milliliter prepared by determining both the percentage parasitemia and the erythrocyte count of the donor mouse and diluting the blood with isotonic saline in proportions indicated by both determinations^8,26,27^. Parasitemia was monitored by standard methods; thin blood smears were made on glass slides, fixed using methanol, and stained using 10% Giemsa stain. The parasitemia was counted using a light microscope (× 100 oil immersion lens) and was determined as the proportion of infected red blood cells observed relative to the total number of cells in several microscopic fields^28^.

### In vivo assessment of suppressive activities of the *S. officinarum* leaf extract and fractions

The suppressive activities of the leaf extract, fractions and chloroquine against *P. berghei* infection in mice were tested using methods previously described^26,27,29^. The study involved fifty-four mice which were inoculated with the parasite on the first day (D_0_) and thereafter divided into nine groups each consisting of six mice. The mice were treated as follows; groups 1–3 were orally administered 170, 340 and 510 mg/kg of crude extract respectively, groups 4–7, were similarly administered 340 mg/kg each of *n*-hexane, DCM, ethyl acetate, and *n*-butanol fractions respectively, infected mice in group 8 received 5 mg/kg of chloroquine orally (positive control) and group 9, which was the negative control group, received 10 mL/kg of distilled water daily for four days (D_0_–D_3_) between 8 and 9 a.m. On the fifth day (D_4_), thin films were prepared from blood collected from the tail of each mouse, which was subsequently stained with 10% Giemsa to reveal parasitized erythrocyte count. The average suppression of parasitemia and percentage chemosuppression were calculated as follows^8,28^.

$$\frac{{({\text{average}}\,\% \,{\text{parasitemia}}\,{\text{positive}}\,{\text{control}} - {\text{average}}\,\% \,{\text{parasitemia}}\,{\text{negative}}\,{\text{control}})}}{{({\text{average}}\,\% \,{\text{parasitemia}}\,{\text{negative}}\,{\text{control}})}} \times 100$$

The mean survival time (MST) of the mice in each treatment group was measured as reported previously^26,27^.

### Determination of in vivo prophylactic activities of the leaf extract and fractions of *S. officinarum*

The extract and fractions were assessed for prophylactic activities using the method earlier described^30^. The mice were treated as follows; groups 1–3 were orally administered 170, 340 and 510 mg/kg of crude extract respectively, groups 4–7, were similarly administered orally 340 mg/kg each of *n*-hexane, DCM, ethyl acetate, and *n*-butanol fractions respectively. Group 8 which was the positive control group was administered 1.2 mg/kg of pyrimethamine and the negative control, group 9 was given 10 mL/kg of distilled water. The mice were treated with the extract and fractions for three consecutive days (D_0_–D_2_), while thin films were prepared from blood collected from the tail of each mouse on the fourth day (D_3_) of the study to assess the parasitaemia level of the mice in each group. The animals were observed for a period of 29 days and mortality in each group was recorded with the date which was used to calculate the MST of the animal^26^.

### Effect of the leaf extract and fractions of *S. officinarum* on established *P. berghei* infection in mice

The curative effects of the extracts, fractions and chloroquine on established *P. berghei* infected mice were investigated using the modified curative model described^27,31^. The 54 mice used in this study were infected intraperitoneally with *P. berghei* on the initial day (D_0_) of the study. Three days after the infection (D_3_), the mice were allocated into 9 groups consisting of 6 mice each. The leaf extract (170, 340, and 510 mg/kg) was respectively given to groups 1–3 orally, 340 mg/kg each of *n*-hexane, ethyl acetate, DCM, and *n*-butanol fractions was respectively administered orally to groups 4–7. Group 8 as the positive control group was orally administered 5 mg/kg chloroquine and group 9, the negative control group was orally given 10 mL/kg distilled water. All treatments, crude extract, fractions and chloroquine, were orally administered once daily for 5 days. Tail blood of each mouse was used to prepare Giemsa-stained thin smears. Tail blood was collected on every treatment day to monitor the parasitaemia. Rectal temperature of individual mouse was monitored and recorded daily throughout the treatment period^32^. The animals were observed for a period of 29 days (D_0_–D_28_) and mortality in each group was recorded with the date which was used to calculate the mean survival time of the animal^8,26,27^.

### Gas chromatography–mass spectrometry analysis

Gas chromatography-mass spectrometry (GC–MS) data of the fractions were recorded on an Agilent 7890A gas chromatograph connected with an Agilent MS model 5975C MSD detector (Agilent Technologies, USA). A HP5-MS column 5% phenyl-methylpolysiloxane, 30 m × 0.25 mm × 0.25 µm was used with a helium gas flow under a pressure of 10 psi. The injector temperature was set at 280 °C. The oven temperature started from 150 °C for 3 min, and increased to 300 °C at 10 °C/min, and held for 5 min at 300 °C. The mass spectrometer was operated using the electron ionization mode at 70 eV. The total extract or fractions were either directly injected in *n*-hexane or after derivatizing to form TMSi derivatives with *N*,*O*-Bis(trimethylsilyl)trifluoroacetamide (BSTFA) as described previously^7,33,34^. The phytochemicals were identified by comparison of spectra in the NIST 2011 database.

### High performance liquid chromatography with ultraviolet–visible spectrophotometry detection (HPLC–UV/vis) analysis

For HPLC–UV/vis analysis of the non-polar DCM fraction, 4 mg/mL of DCM fraction of sugarcane was prepared in methanol and 20 µL was injected into the HPLC with an analytical HPLC column coupled with a UV–Vis detector (Phenomenex, UK; 5 µm particle size, 4.6 × 250 mm). The mobile phase used two solvent systems. Solvent A consisted of water with 0.1% TFA, and solvent B was 100% methanol. The mobile phase calibration started with 50% B and rose to 100% B over 25 min and was maintained at 100% B for 6 min at a flow rate of 1 mL/min at 215 nm and 254 nm absorbance. For the HPLC analysis of the more polar butanol fraction, 4 mg/mL of the butanol fraction of sugarcane was prepared in methanol. 20 µL of the extract was injected into the HPLC for analysis. The mobile phase used two solvent systems. Solvent A consisted of water with 0.1% TFA and solvent B was 100% methanol. The mobile phase calibration was maintained at 100% A for 10 min and solvent B rose to 60% over 30 min. Furthermore, B rose to 100% in 10 s and was maintained at 100% for 10 min at a flow rate of 1 mL/min at 254 nm and 350 nm absorbance.

### Liquid chromatography time of flight mass spectrometry (LC-TOF–MS) analysis

An Agilent Infinity 1260 series system comprising a G1316A TCC Thermostated column compartment, G1312B Binary Pump, G4225A Hip Degasser, G1329B ALS Auto sampler and Agilent 6530B Q-TOF mass spectrometer (Agilent, Santa Clara, CA, USA) was used for the analysis of DCM and butanol fraction of sugarcane as described previously^7,35^. Further LC-Q-ToF–MS/MS was performed with an electrospray ionization (ESI) ion source using the auto MS/MS feature in the MassHunter software (Agilent Technologies).

The MS detection parameters were set as follows: drying gas temperature, 300 °C; drying gas (N2) flow rate, 11.0 L/min; fragmentor voltage, 125 V; nebulizer, 50 psig and capillary, 4000 V; skimmer, 65 V; Oct RF Vpp, 750 V. The sample collision energy was set at 15 and 40 V. All acquisition and analysis of data were controlled by MassHunter software (Agilent Technologies). Mass spectra were recorded in the range of *m/z* 100–1000 in MS and 100–800 for MS/MS with accurate mass measurement of all mass peaks. Each sample was analyzed in both positive and negative modes to give abundant information for structural identification.

An external calibration solution (Agilent calibration solution) was continuously sprayed in the ESI source of the Q-TOF system, employing the ions with *m/z* 112.9855 (TFA anion) and 1033.9881 [HP-0921 (TFA adduct)] in negative mode and *m/z* 121.0509 (purine) and *m/z* 922.0098 (HP921) in positive mode to recalibrate the mass axis, ensuring mass accuracy and reproducibility throughout the chromatographic run.

1–2 µL of DCM and butanol fractions dissolved in 0.1% formic acid in methanol were injected into a narrow bore LC column (Infinity Lab Poroshell 120 EC-C18, 2.1 × 100 mm, particle size, 2.7 µm, Thermo Fisher Scientific, UK) coupled with A detector with mass range of 100–1000 *m/z*. For analysis of DCM fraction, a flow rate of solvent was set at 0.3 mL/min by increasing from 50 to 100% B over 25 min and then keeping for 100% B for 6 min. Solvent A and B are 0.1% formic acid in water and methanol, respectively. For analysis of the butanol fraction a flow rate of solvent was set at 0.3 mL/min by remaining 0% B over 10 min and increasing to 60% (from 10 to 50 min) and maintaining 100% B for 10 min. For annotation of compounds, the raw .d data files were converted to .abf files using Reifycs Analysis Base File Converter (https://www.reifycs.com/AbfConverter/), which were then carried out using MS-DIAL ver.4.80 (Riken, Osaka University, Japan)^36^ with MS-Dial metabolomics MPS spectral databases (available at:http://prime.psc.riken.jp/compms/msdial/main.html; last updated on 19 April 2022).

### Nuclear magnetic resonance (NMR) spectroscopy analysis

^1^H NMR (400 MHz) spectroscopy and heteronuclear single quantum coherence spectroscopy (HSQC) analysis of the DCM fraction (CDCl_3_) and butanol fraction (DMSO-d_6_) were recorded using a Brucker 400 MHz NMR instrument.

### Statistical analysis

Values are represented as mean ± standard deviation (SD) or standard error of mean (SEM) and significance relative to control were considered at p ˂ 0.05. Data collected in this study were analysed using one-way ANOVA followed by a post-hoc Tukey Kramer multiple comparison test using GraphPad Prism software Inc. (5.0, La Jolla, CA, USA).

### Declaration

The animal study is reported in accordance with ARRIVE guidelines (https://arriveguidelines.org).

## Results

### In vitro antiplasmodial activity of *S. officinarum* fractions against *P. falciparum* Dd2^luc^

**Figure 1:**
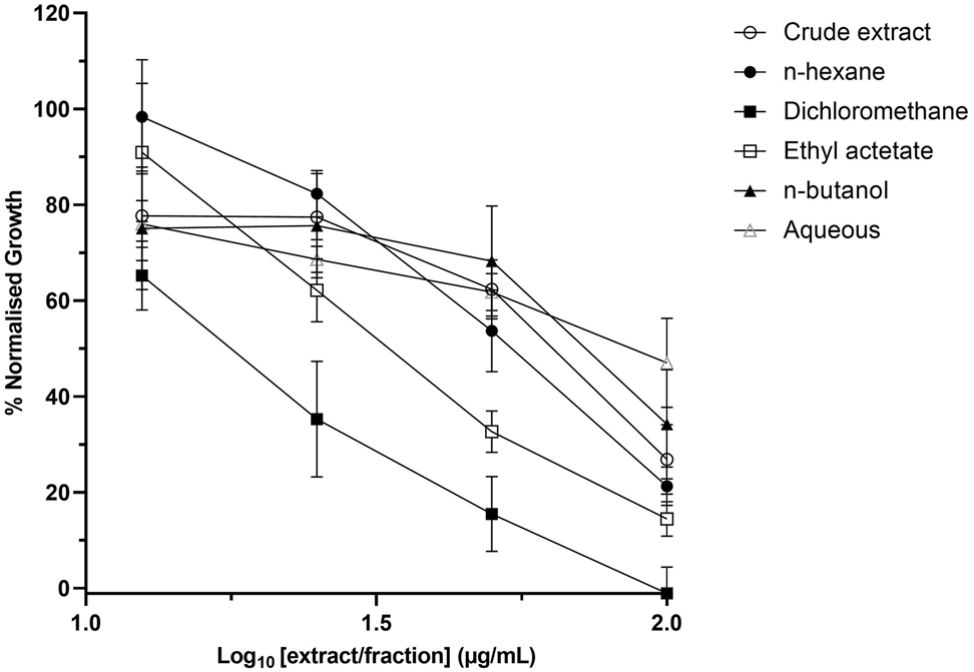
In vitro antiplasmodial activity of sugarcane leaf extracts and fractions against *P. falciparum Dd2*^*luc*^*.* Normalised growth of parasites exposed to 100, 50, 25 and 12.5 µg/mL of crude extract, *n*-hexane, DCM, ethyl acetate, *n*-butanol and aqueous fractions. Data presented as mean ± standard deviation of normalised growth (n ≥ 3) versus concentration of extract/fraction.

**Figure 2:**
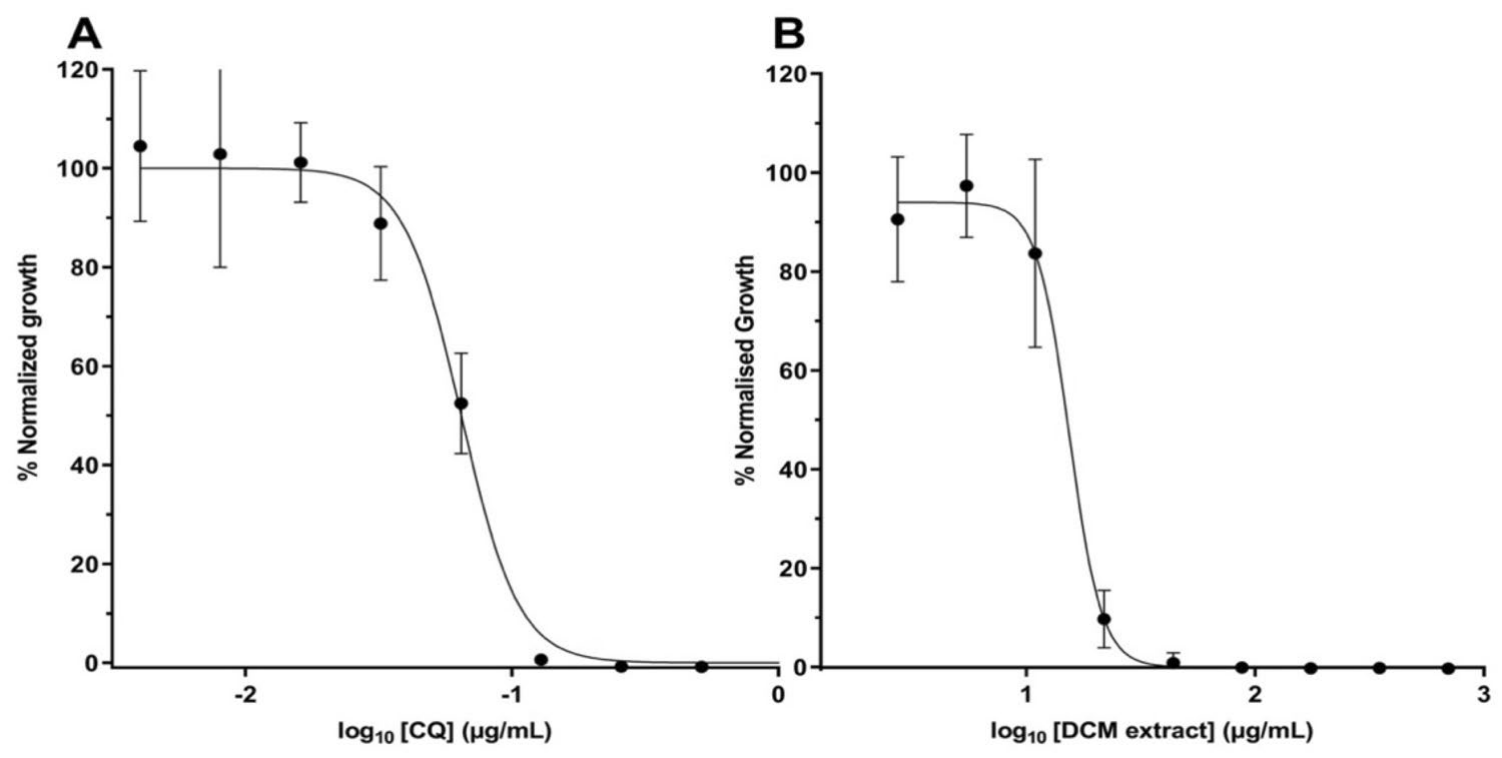
Determination of the EC_50_ of the dichloromethane (DCM) fraction of sugarcane leave extract. Normalised growth of parasites exposed to (**A**) chloroquine (CQ) and (**B**) DCM fraction. Data presented as mean ± standard deviation of normalised growth (n ≥ 6) versus concentration of the fraction.

To provide an initial characterization of the in vitro antiplasmodial activity of the leaf extract and fractions, *P. falciparum* strain Dd2^luc^ were exposed to a limited two-fold dilution (100–12.5 µg/mL) for 48 h. Normalised growth, compared to untreated control, was plotted against concentration of extract/fraction (Fig. 1). The DCM, ethyl acetate and *n*-hexane extracts were all more potent than the unfractionated total extract. Interestingly, the aqueous extract was least potent. To estimate EC_50_, nine-point two-fold dilutions (from 700 to 1.4 µg/mL) were next tested. Using this same approach (although starting from 1 µg/mL), the EC_50_ of a standard reference, chloroquine, was also determined. As expected, the EC_50_ of chloroquine was estimated at 0.065 µg/mL (Fig. 2). The EC_50_ of the most potent, DCM, extract was estimated as 15.4 µg/mL, with those for the ethyl acetate and *n*-hexane fraction (33.2 and 54.4 µg/mL, respectively) following the trend demonstrated from the preliminary analysis (Table 1). The range of EC_50_ of the total extract and the *n*-butanol and aqueous fraction were estimated (Table 1) with the aqueous fraction least activity.

**Table 1:** In vitro antiplasmodial activity of *S. officinarum* extracts and fractions against chloroquine resistant (Dd2) strains of *P. falciparum.*

*S. officinarum* fraction	IC_50_ (µg/mL)	95% confidence intervals
Crude extract	> 55	ND
*n*-Hexane fraction	54.4*	45.4–66.2
DCM fraction	15.4*	14.1–16.8
Ethyl acetate fraction	33.2*	22.6–52.3
*n*-Butanol fraction	> 55	ND
Aqueous fraction	> 100**	ND
Chloroquine	0.065	0.06–0.07

Values expressed as IC_50_ (µg/mL) ± standard deviation (n =  ≥ 6). *ND* not determined. *Estimated IC_50_ using GraphPad prism. **IC_50_ greater than the highest concentration tested.

### Determination of median lethal dose (LD_50_)

To choose the appropriate doses of the extract and fractions for evaluation of their in vivo antimalarial activity, the median lethal dose (LD_50_) of the total extract was determined as 1732 mg/kg. Therefore, lower doses such as 170, 340, and 510 mg/kg were used for the following in vivo antimalarial testing.

### In vivo chemosuppressive effect of ethanol leaf extract and fractions of *S. officinarum* on 4-day mice test

Based on the in vitro results, all fractions with an EC_50_ determined to be less than 100 µg/mL were further evaluated for their in vivo potential^37^. The 4-day suppressive test is intended to evaluate the antimalarial activity of the extract/fractions on early periods of infection^8^. Characterisation of suppressive activities of the leaf extract and its fractions showed that all extracts (170–510 mg/kg) caused a dose-dependent decrease in parasitaemia in the various groups of treated mice. The observed reductions were statistically significant when compared to the negative control group (*p* < 0.001).The *n*-butanol fraction was the most active in this suppressive test with a chemosuppression of 68.4% and MST value of 26.0 days although lower compared to that of the standard drug, chloroquine, 5 mg/kg (93.9%) was given to mice in positive control group (Table 2).

**Table 2:** Suppressive activities of leaf extract and fractions of *S. officinarum* during early *P. berghei* infection in mice.

Treatment	Dose (mg/kg)	Parasitaemia	Chemosuppression (%)	Mean survival time (MST) (day)
Control	Water	34.2 ± 1.1		14.0 ± 1
Crude extract	170	21.4 ± 2.2^c^	37.6	18.7 ± 0.9^a^
	340	13.1 ± 1.7^c^	61.6	23.3 ± 1.1^c^
	510	10.2 ± 2.1^c^	70.2	27.7 ± 1.3^c^
*n-*Hexane fraction	340	12.4 ± 1.1^c^	63.7	25.0 ± 1.2^b^
DCM fraction	340	14.6 ± 1.6^c^	57.4	20.3 ± 1.0^b^
Ethyl acetate fraction	340	12.9 ± 2.1^c^	62.4	21.7 ± 1.0^c^
*n*-Butanol fraction	340	10.8 ± 1.8^c^	68.4	26.0 ± 1.0^b^
Chloroquine	5	2.1 ± 1.4^c^	939	30.0 ± 0.0^c^

Values are expressed as mean ± SEM. Significant relative to control. ^a^p < 0.05; ^b^p < 0.01; ^c^p < 0.001 (n = 6).

### In vivo prophylactic activities of *S. officinarum* leaf extract and fractions

After extract treatment of the infected mice with three doses, their parasitaemia levels reduced significantly (*p* < 0.01–0.001) and dose-dependently (Table 3). Among all fractions using the same dose of 340 mg/kg, the *n*-butanol fraction which exerted the highest prophylactic potential exhibited a chemosuppressive effect of 39.2% and MST value of 17.0 days. This was low compared to that exhibited by the standard drug, pyrimethamine 1.2 mg/kg but statistically significant (*p* < 0.001) when compared to the negative control group (Table 3).

**Table 3:** Prophylactic activities of leaf extract and fractions of *S. officinarum.*

Treatment	Dose (mg/kg)	Parasitaemia	Chemosuppression (%)	Mean survival time (MST) (day)
Control	–	18.8 ± 1.2	–	10.3 ± 0.3
Crude extract	170	15.5 ± 1.6^b^	17.8	11.7 ± 0.3
	340	15.1 ± 1.2^b^	19.6	12.7 ± 0.7
	510	15.2 ± 2.1^b^	19.3	12.7 ± 0.7
*n-*Hexane fraction	340	14.6 ± 1.3^c^	22.3	13.3 ± 1.9
DCM fraction	340	14.1 ± 2.1^c^	25.1	14.0 ± 1.0
Ethyl acetate fraction	340	13.0 ± 0.8^c^	31.0	15.3 ± 0.3
*n*-Butanol fraction	340	11.5 ± 1.3^c^	39.2	17.0 ± 1.0^a^
Pyrimethamine	1.2	2.2 ± 1.0^c^	88.6	25.0 ± 0.3^c^

Values are expressed as mean ± SEM. Significance relative to control. ^a^p < 0.05; ^b^p < 0.01; ^c^p < 0.001 (n = 6).

### In vivo curative effect of *S. officinarum* leaf extract and fractions on established *P. berghei* infection in mice

All leaf extract and fractions exhibited a dose-dependent reduction of parasitaemia levels when compared to that of negative control group (Table 3). The highest curative effect was exhibited by *n*-butanol fraction with a chemosuppressive effect of 66.9%, which was lower when compared to that exhibited by the standard drug, chloroquine (100%) administered to the positive control group.

The leaf extract and fractions were observed to further offer prominent protection to the treated mice as demonstrated in the statistically significant (p < 0.05–0.001) MST of the treated animals. *P. berghei* infected mice treated with *n*-butanol fraction were observed to survive significantly (*p* < 0.05) longer than mice in other groups with a mean survival time, 23.0 ± 1.1 days followed by DCM fraction treated mice, with a mean survival time of 20.8 ± 0.8 days. However, the mean survival times of the extracts/fractions-treated groups were significantly (*p* < 0.05) less than that of mice treated with the standard drug, chloroquine (28.5 ± 1.2 days; Table 4).

**Table 4:** Mean survival time of mice treated with leaf extract and fractions of *S. officinarum* during established *P. berghei* infection in mice.

Treatment	Dose (mg/kg)	Mean survival time (days)	% Parasitaemia on D7	Chemosuppression (%)
Control	–	12.5 ± 0.3	31.7 ± 2.4	–
Crude extract	170	18.3 ± 0.8^b^	14.5 ± 1.6^c^	54.1
	340	19.5 ± 1.0^c^	13.7 ± 1.3^c^	56.8
	510	21.3 ± 1.5^c^	11.5 ± 2.78 ^c^	63.9
*n-*Hexane fraction	340	19.8 ± 0.8^c^	15.0 ± 1.86^b^	52.6
DCM fraction	340	20.8 ± 0.8^c^	12.2 ± 1.3^c^	61.5
Ethyl acetate fraction	340	20.0 ± 0.0^c^	12.5 ± 0.9^c^	60.7
*n*-Butanol fraction	340	23.0 ± 1.1^c^	10.5 ± 2.4^c^	66.9
Chloroquine	5	28.5 ± 1.2^c^	1.1 ± 0.9^c^	96.7

Values are expressed as mean ± SEM. Significance relative to control. ^a^p < 0.05; ^b^p < 0.01; ^c^p < 0.001. n = 6.

### Effect of *S. officinarum* leaf extract/fractions on rectal temperatures of *P. berghei*-infected mice

Treatment of *P. berghei*-infected mice with *S. officinarum* leaf extract/fractions did not exert any significant (*p* > 0.05) effect on the rectal temperatures of the treated mice when compared with that of control on days 5 and 7 (Table 5).

**Table 5:** Effect of leaf extract and fractions of *S. officinarum* on rectal temperatures of mice infected with *P. berghei* during established infection.

Treatment	Dose (mg/kg)	Rectal temperature (°C)		
		D0	D3	D7
Control	–	34.9 ± 0.5	35.2 ± 0.02	35.8 ± 0.1
Extract	170	34.7 ± 0.1	35.4 ± 0.10	35.8 ± 0.1
	340	35.1 ± 0.3	35.3 ± 0.02	35.7 ± 0.1
	510	34.7 ± 0.2	35.3 ± 0.1	35.8 ± 0.1
*n-*Hexane fraction	340	34.9 ± 0.3	35.3 ± 0.1	35.8 ± 0.1
DCM fraction	340	34.4 ± 0.1	35.3 ± 0.1	35.7 ± 0.04
Ethyl acetate fraction	340	35.0 ± 0.1	35.1 ± 0.03	35.7 ± 0.1
*n*-Butanol fraction	340	35.0 ± 0.2	35.3 ± 0.1	35.8 ± 0.1
Chloroquine	5	35.2 ± 0.3	35.4 ± 0.1	35.2 ± 0.1

Values are expressed as mean ± SEM, n = 6.

### GC–MS analysis

**Figure 3:**
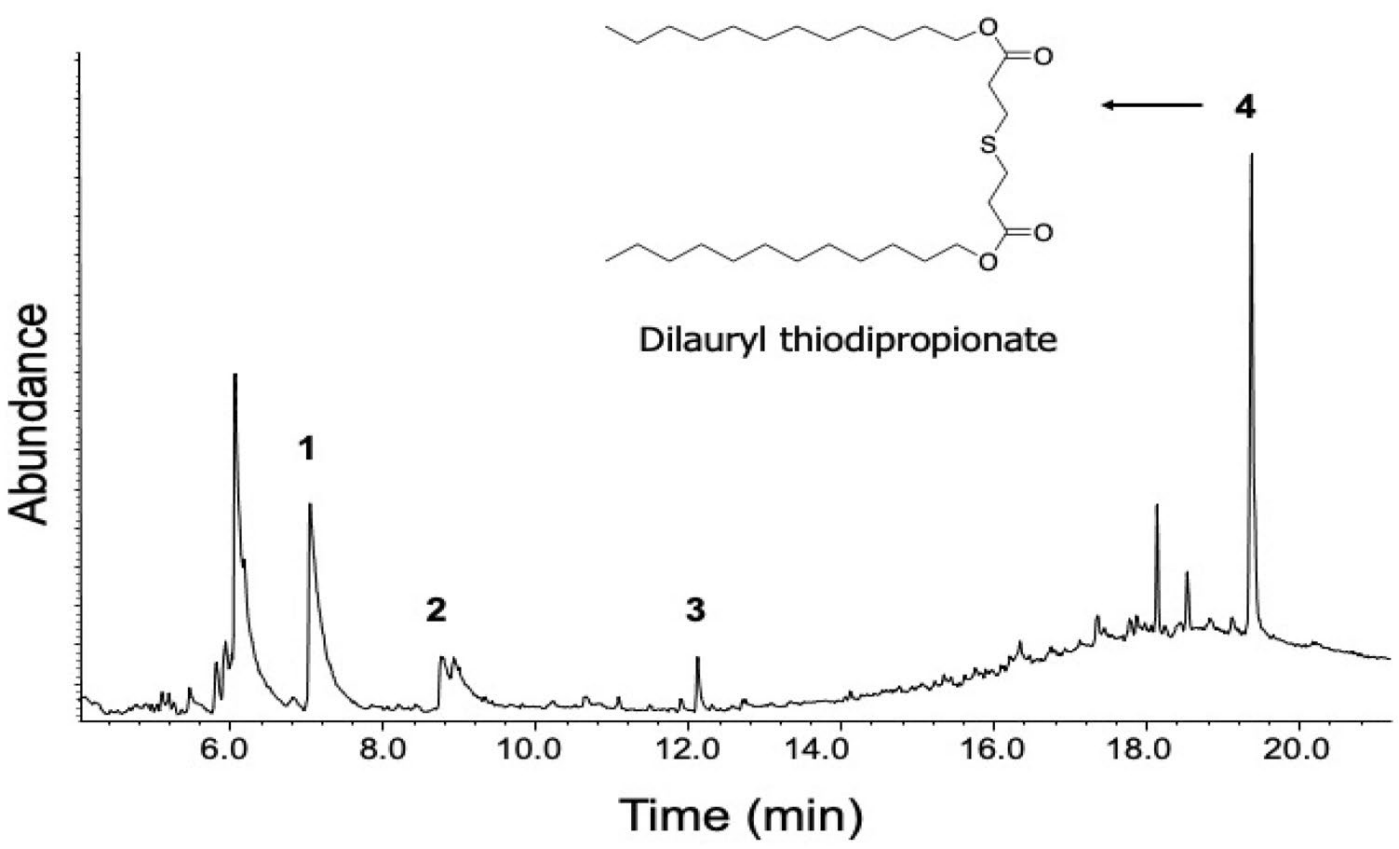
GC–MS chromatogram of DCM fraction of *S. officinarum.* Four compounds are identified with the chemical structure of compound **4** indicated.

GC–MS analysis of the most active (in vitro) DCM fraction showed it consisted of an unusual sulphur-containing compound dilaurylthiodipropionat (Supplementary Fig. S1), *n*-hexadecanoic acid, octadec-9-enoic acid, and bis(2-ethylhexyl) phthalate (Fig. 3 and Table 6, Supplementary Table S1). GC–MS analysis of the most potent (in vivo) *n*-butanol fraction after TMSi derivation indicated the presence of phenolic acids such as 3,4,5-trihydroxy benzoic acid (gallic acid) and 4-hydroxycinnamic acid as well as *n*-hexadecanoic acid, β-sitosterol, and others (Table 7, Supplementary Fig. S2). GC–MS analysis of the total crude extract after TMSi derivation (Supplementary Table S2), and other less active fractions including *n*-hexane (Supplementary Fig. S3, Supplementary Table S3) and ethyl acetate fractions (Supplementary Table S4) revealed the presence of many more compounds such as tocopherol, squalene, and octadecanoic acid among others.

**Table 6:** GC–MS analysis of the dichloromethane (DCM) fraction of *S. officinarum.*

Peak	Rt (min)	Compound name	Mol. Formula	Monoisotopic Mol. Mass	Match quality (%)
1	7.126	*n*-Hexadecanoic acid	C_16_H_32_O_2_	256.24	98
2	8.773	Octadec-9-enoic acid	C_18_H_34_O_2_	282.26	96
3	12.123	Bis(2-ethylhexyl) phthalate	C_24_H_38_O_4_	390.28	93
4	19.342	Propanoic acid, 3,3′-thiobis-, didodecyl ester (Dilaurylthiodipropionate)	C_30_H_58_O_4_S	514.41	95

**Table 7:** GC–MS analysis of the *n*-butanol fraction of *S. officinarum after* TMSi derivation.

Peak	Rt	TMSi derivatives	Mol. Formula	Mol. Mass	Parent compounds in *n*-butanol fraction
1	6.378	4-Hydrocinnamic acid, p-(trimethylsiloxy)-, trimethylsilyl ester	C_15_H_26_O_3_Si_2_	310.14	4-Hydroxycinnamic acid
2	7.115	Benzoic acid, 3,4,5-tris(trimethylsiloxy)-, trimethylsilyl ester	C_19_H_38_O_5_Si_4_	458.18	3,4,5-Trihydroxy benzoic acid (Gallic acid)
3	7.250	α-d-Xylopyranose, 1,2,3,4-tetrakis-*O*-(trimethylsilyl)-	C_17_H_42_O_5_Si_4_	438.21	α-d-Xylopyranose
4	7.543	3-Hydroxy-3-(4′-hydroxy-3′-methoxyphenyl)propionic acid, tri-TMS	C_19_H_36_O_5_Si_3_	428.19	3-Hydroxy-3-(4′-hydroxy-3′-methoxyphenyl)propionic acid
5	7.782	Hexadecanoic acid, trimethylsilyl ester	C_19_H_40_O_2_Si	328.28	Hexadecanoic acid
6	8.344	Octadecane-1,2-diol, bis(trimethylsilyl) ether	C_24_H_54_O_2_Si_2_	430.37	Octadecane-1,2-diol
7	18.326	β-Sitosterol trimethylsilyl ether	C_32_H_58_OSi	486.43	β-Sitosterol

### HPLC UV/Vis analysis

HPLC UV/Vis analysis of the DCM fraction with UV absorption at 215 and 254 nm indicated the presence of a large number of metabolites. Further HPLC analysis of the *n*-butanol fraction at 254 and 350 nm indicated the presence of phenolic compounds likely flavonoids with stronger absorbance at 350 nm (Supplementary Fig. S4).

### LC-TOF–MS analysis

**Figure 4:**
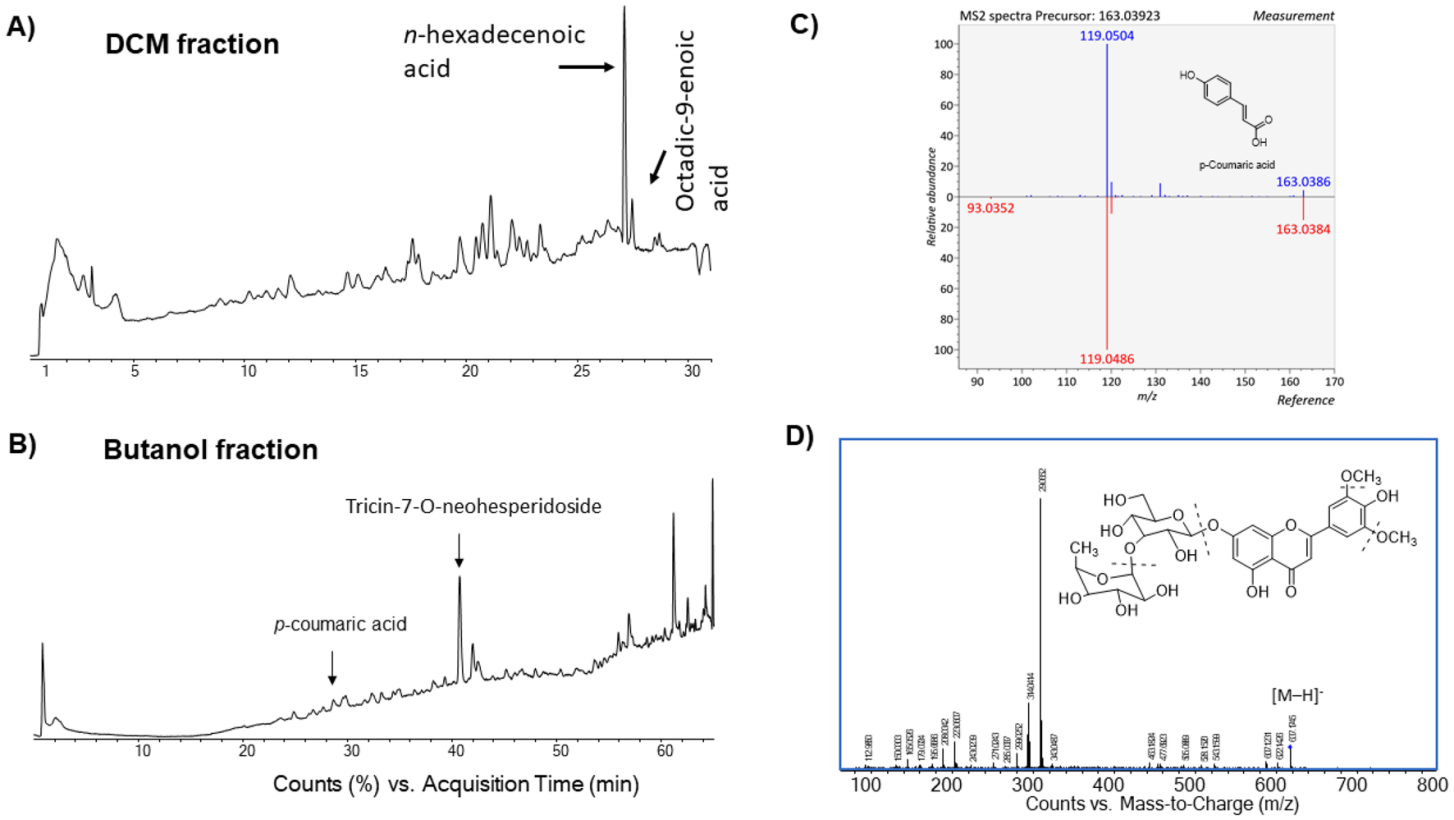
LC-TOF-(negative mode) MS/MS analysis of the DCM (**A**) and butanol (**B**) fractions of sugarcane leave extracts. (**C**) Exemplar tentative identification of *p*-coumaric acid (4-hydroxycinnamic acid) from the butanol fraction via MS-Dial tool. Comparison of CID MS/MS spectrum of *p*-coumaric acid (25 V) with that of reference spectrum in the library. (**D**) Tentative identification of tricin-7-*O*-eohesperoside from the butanol fraction via a combination of MS-Dial tool and local sugarcane compound database. CID-MS/MS spectrum (40 V) of mass 637.1784 indicating a major fragment ion at *m/z* 329.0676 through the loss of rhamnose and glucose. Initial loss of rhamnose (− 146) was also observed on the positive CID-MS/MS (15 V) spectrum (Supplementary Fig. S5) in agreement with data in literature^38^.

LC–TOF-MS/MS analysis of the DCM fraction indicated the presence of a number of fatty acids such as oxidized fatty acids, hexadecenoic acid and a series of unsaturated fatty acids (Fig. 4A, Table 8).

**Table 8:** LC-TOF–MS analysis of the DCM fraction of *S. officinarum* and tentative identification of compounds.

Compounds	Rt (min)	Negative ESI (found)	Mass difference (mDa)	Mol. formula	MS/MS
Azelaic acid	2.731	187.0974	0.14	C_9_H_16_O_4_	187.0970, 171.1029, 143.1108, 125.0974 (100%)
Oxidized fatty acid	12.257	305.1765	3.16	C_18_H_26_O_4_	305.1753, 249.1508, 209.1225, 174.9574, 135.0826, 112.9861
Alkyl-phenylketone	14.650	253.1215	0.14	C_17_H_18_O_2_	253.1221, 191.9451, 112.9834
Oxidized fatty acid	15.147	307.1922	1.28	C_18_H_28_O_4_	307.1922, 291.1927, 265.1811, 225.1509, 174.9578, 151.1118, 112.9854
Oxidized fatty acid	16.632	309.2081	1.74	C_18_H_30_O_4_	309.2062, 291.195, 225.1497, 197.1182, 171.1035, 137.0980, 112.9858
Oxidized fatty acid	19.948	293.2127	1.04	C_18_H_30_O_3_	293.2115, 275.1992, 223.1332, 195.1380, 174.9552, 112.9859
Oxidized fatty acid	21.359	295.2273	0	C_18_H_32_O_3_	295.2278, 277.2170, 249.2228, 195.1393, 171.1036, 112.9887
Oxidized fatty acid	22.135	295.2282	0.99	C_18_H_32_O_3_	297.2429, 171.1003, 112.9864
Oxidized fatty acid	22.982	297.2432	0.76	C_18_H_34_O_3_	297.2429, 174.956, 112.9864
Oxocins	23.617	299.2598	2.5	C_16_H_28_O_5_	299.2592, 174.9554, 112.9859
Linoleic Acid	26.651	279.2329	0.03	C_18_H_32_O_2_	279.2592, 241.2167, 158.9795 112.9871
*n*-Hexadecenoic acid (palmitic acid)	27.113	255.2332	0.2	C_16_H_32_O_2_	255.2332, 187.1302 112.9838
Octadic-9-enoic acid (oleic acid)	27.780	281.2491	0.48	C_18_H_34_O_2_	281.2487, 255.2321, 157.0115, 112.9859

LC-TOF–MS/MS analysis of the butanol fraction indicated the presence of a phenolic acid, 4-hydroxycinnamic acid (Fig. 4B,C), a flavonoid, 3,4′,5,6,7-pentamethoxyflavone^39^, and a flavonoid glycoside, tricin-7-*O*-neohesperidoside^38,40^ (Fig. 4D and Supplementary Fig. S5) and and an oxidized fatty acid (Table 9).

**Table 9:** LC-TOF–MS analysis of the butanol fraction of *S. officinarum* leaves and tentative identification of compounds.

Compounds	Rt (min)	Negative ESI (found)	Mass difference (Da)	Mol. formula	MS/MS
4-Hydroxycinamic acid (*p*-Coumaric acid)	28.056	163.0393	0.84	C_9_H_8_O_3_	163.0386, 130.9666, 119.0504 (100%), 112.9864
5,9-Dihydroxy-7-(hydroxymethyl)-5,7-dimethyl-4,5a,6,8,8a,9-hexahydro-1H-azuleno[5,6-c]furan-3-one	28.550	281.1388	0.64	C_15_H_22_O_5_	281.1361, 251.1160, 237.1485, 189.1250, 171.1205
3′,4′,5′,5,7-Pentamethoxyflavone	32.077	371.11594	2.32	C_20_H_20_O_7_	371.1171, 353.1004, 341.1028, 327.1277, 309.1102, 294.0852, 248.9557, 154.9724
2,3,8,9,10-Pentamethoxy-6a,11a-dihydro-6H-[1]benzofuro[3,2-c]chromene (Pterocarpans)	40.332	373.1300	0.8	C_20_H_22_O_7_	343.1186, 204.9683, 154.9741
Tricin-7-*O*-neohesperidoside	42.025	637.1782	4.27	C_29_H_34_O_16_	637.1784, 555.9921, 329.0676, 223.0612
9,12,13-Trihydroxy-10,15-octadecadienoic acid	54.371	327.21942	3.11	C_18_H_32_O_5_	327.2165, 309.2018, 291.1929, 280.9822, 229.1430, 211.1315, 171.1005, 112.9844

### NMR analysis

**Figure 5:**
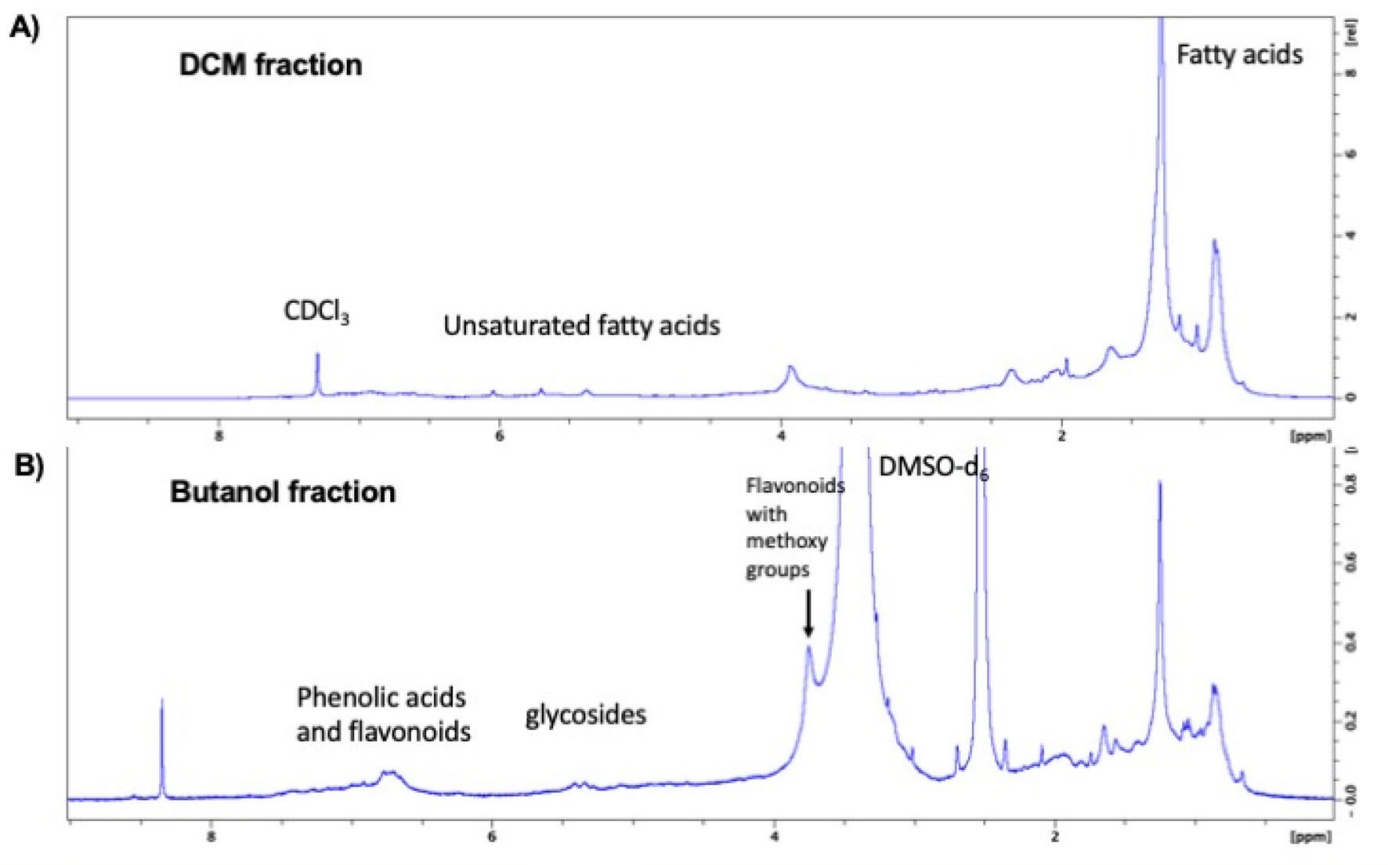
^1^H NMR (400 MHz) analysis of the DCM fraction in CDCl_3_ (**A**) and the butanol fractions in DMSO-d_6_ (**B**) of sugarcane leaf extracts.

^1^H NMR analysis of the DCM fraction and the butanol fraction further supported the presence of fatty acids and phenolic acids/flavonoid glycosides, respectively (Fig. 5). HSQC of the DCM fraction showed the crossing peak between 29.1 (^13^C) and 1.25 (^1^H) ppm, evidenced for long chain fatty acids (Supplementary Fig. S6). HSQC of the butanol fraction showed the crossing peak between 56.6 ppm (^13^C) and 3.72 ppm (^1^H), evidenced for the methoxy groups likely for 3,4′,5,6,7-pentamethoxyflavone^39^ and tricin-7-*O*-neohesperidoside^41^ (Supplementary Fig. S7).

## Discussion

*Saccharum officinarum* leaves are used in traditional medicines to treat malaria infection in Ibibio land of Southern Nigeria as well as in the Dangme West District of Ghana^12^. This work set out to validate and authenticate these antimalarial claims and explore the potential for discovery of novel potent antimalarial compounds. *Saccharum officinarum* leaf extract and fractions were tested for in vitro antiplasmodial activity against the *P. falciparum* (Dd2) strain. In vitro antiplasmodial evaluation of the leaf extract and fractions against *P. falciparum* revealed that *S. officinarum* leaf extract and fractions exhibit low to moderate antiplasmodial activity, with the DCM fraction (IC_50_ = 15.4 µg/mL) exerting the highest effect on the parasites followed by the ethyl acetate fraction whilst the aqueous fraction displayed the least activity with its IC_50_ larger than 100 µg/mL. We set a cut-off activity at 100 µg/mL for validating the in vivo efficacy by considering the low toxicity profile of the total extract with LD_50_ of 1.7 g/kg/day and wide availability of sugarcane leaves. However, we acknowledge that this cut-off EC_50_ is higher than those often regarded as having very good or good antimalarial activity (5–10 µg/mL)^42^. Further in vivo activity of all other fractions and total extracts against *P. berghei* infection in mice was evaluated using suppressive, curative, and prophylactic antimalarial models, which are the standard methods often used for validating antimalarial candidates^27^. The results of this study showed that *S. officinarum* leaf extract and fractions caused good or moderate reduction of parasitaemia in treated mice in the three models tested dose-dependently with *n*-hexane and *n*-butanol fractions exerting more significant and good in vivo activity according to the set criteria^42^. The in vivo anti-malarial activity of an extract is considered as very good, good and moderate if the suppression is ≥ 50% at 100 mg/kg, 250 mg/kg and 500 mg/kg body weight/day, respectively^42^. The leaf extract and fractions offered a degree of protection to the treated mice as observed in the significant prolonged MST of the treated mice. This may have resulted from suppression of parasite growth or schizonticidal activity of the leaf extract and fractions. Furthermore, the rectal temperature was measured to assess the effect of the extract/fractions on temperature of the infected mice. This was done in order to assess their potential clinical use as fever is a symptom of malaria in humans in addition to parasitological cure^32^. However, no significant effect (Table 5) was observed which may have been as a result of the short duration of the study, which agrees with previous report that *P. berghei* does not cause fever or increase in temperature^32^. Together, these data authenticate the antimalarial potential of the leaf extract. These data suggest the presence of the active antiplasmodial compound(s) in these fractions.

Whilst the DCM and ethyl acetate fractions were more active in vitro against *P. falciparum*, the *n*-hexane and *n*-butanol fractions were more active against *P. berghei* in the in vivo study. This unexpected outcome is likely a result of the use of different parasite species in the two systems tested as well as the potential involvement of immune system activity in vivo which may have been stimulated by the phytochemical constituents of the fractions applied^43^. Immunostimulatory potentials of tannins, squalene, β-sitosterol and fatty acids such as linoleic acids have been documented^44,45^. The presence of these compounds in the leaf extract and fractions may have contributed to a potential immunostimulatory effect. Negative in vitro results or high in vitro EC_50_ values of extract and fractions as observed in this study with *n*-hexane and *n*-butanol fractions do not invalidate the in vivo antimalarial activities of these extract/fractions. The in vitro models for screening antimalarial agents are known to have some limitations, of potential significance here in that due consideration are not given to pro-drug effect, selective accumulation and activity of immune system in the prevention and control of infections^43^.

Here, chemical profiling of the extracts/fractions was carried out by a combination of GC–MS, HPLC–UV–VIS, LC-TOF–MS/MS, 1D and 2D NMR techniques to reveal the bioactive compounds. Metabolites such as phenolics, flavonoids, terpenes, β-sitosterol and polyunsaturated fatty acids (PUFAs) among others have been revealed by comprehensive analysis of the extract and fractions. *n*-Hexadecanoic acid and octadec-9-enoic acid in the DCM fraction were detected by both GC–MS and LC–MS analysis, while propanoic acid, 3,3′-thiobis-, didodecyl ester (dilaurylthiodipropionate) was only detected by GC–MS but not by LC–MS probably due to its highly hydrophobic nature. The simple phenolic acid 4-hydroxycinnamic acid was detected by both GC–MS and LC–MS in the butanol fraction. Further LC–MS/MS analysis of this fraction indicated the presence of flavonoid and flavonoid glycoside such as tricin-7-*O*-neohesperidoside^19^ (Fig. 4D and Supplementary Fig. S5), which was previously reported from the sugarcane extracts^13,38^. The existence of these compounds were further supported by 1D and 2D NMR spectroscopic analysis. However, absolute identification of plant metabolites in a complex mixture of extract is very challenging when standard compounds are not available in this case to directly compare their EI-MS or MS/MS spectra and retention times.

The presence of these compounds in the leaf extract/fractions could have contributed to the observed antimalarial activities in this study. There are reports of the involvement of some secondary metabolites of plants such as flavonoids and triterpenoids and PUFAs such as 9-octadecenoic acid methyl ester, 9,12-octadecadienoic acid methyl ester, and 9,12,15-octadecatrienoic acid in antiplasmodial activities of plants^2^. The antiplasmodial activity of PUFAs are reported to correlate with the number of unsaturation bond^46–48^. Likewise, β-sitosterol present in the extract/fractions has been reported to exert antiplasmodial activity^49^. Interestingly, an unusual thio-containing dilaurylthiodipropionate has been identified from the sugarcane leaves for the first time. It was previously only found in *Hystrix Brachyura* Bezoar extract^50^. Gallic acid and other phenolic acids have been identified in the *n*-butanol fraction of sugarcane consistent with previous findings^13^. Antiplasmodial activity of gallic acid has been previously reported^7^. Flavonoids and flavone glycosides commonly found in sugarcane^13^, are reported to exert antiplasmodial activity^51,52^. Antioxidant activities of phytochemical compounds have been implicated in antiplasmodial activities of many plants. For example, antioxidant potentials of flavonoids have been suggested to be the mechanism of its antiplasmodial activity^53^, since raised free radical levels resulting from *Plasmodium species* infections are implicated in severe malaria complications. Potent antioxidant compounds such as squalene, a triterpene, hexadecanoic acid and β-sitosterol^54^, have been revealed to be present in the extract and fraction by GC–MS and/or LC–MS analysis. There is a likelihood that these compounds found to be present in the extract and active fraction may have contributed to the observed antiplasmodial activities in this study. Scavenging of these free radicals could be suggested to be one of the modes of antiplasmodial activity of this extract. Besides antioxidant activity, flavonoids exert antiplasmodial effect through other mechanisms such as stimulation of immune system and inhibition of fatty acid synthesis in the parasite^55^ and inhibition of protein synthesis^56^. The leaf extract/fractions may be exerting its antiplasmodial activity through one of these mechanisms. The presence of these compounds in the leaf extract and fractions may have contributed to the schizonticidal activity.

The data obtained from this study suggest that *S. officinarum* leaf extract and fractions possess antimalarial activity and identify some phytochemical constituents, which may contribute to the observed biological activity. This confirms and authenticates its use.

## Supplementary Information

Supplementary Information.

## Data Availability

All data generated or analysed during this study are included in this published article and its supplementary information files.
