# Supplementary material for: In vitro and in vivo antimalarial activity and chemical profiling of sugarcane leaves
Source: Sci Rep. 2022 Jun 17;12:10250. doi: 10.1038/s41598-022-14391-8 (PMC9205285; doi:10.1038/s41598-022-14391-8)
Supplement: Supplementary file 1 — Supplementary Information. [file 41598_2022_14391_MOESM1_ESM.pdf]

Scientific Reports

Supplementary materials

In vitro and *in vivo* Antimalarial Activity and Chemical Profiling of Sugarcane Leaves

Jude E. Okokon[1,*], Rebecca Mobley[2], Utibe A. Edem[3], Augustine I. Bassey[3], Idowu Fadayomi[4], Falko Drijfhout[5], Paul Horrocks[2], Wen-Wu Li[4,*]

Table S1: GC-MS analysis of DCM fraction of *S. officinarum* after TMSi derivation

PEAK	RT (min)	COMPOUND NAME	FORMULA	MOL. MASS
**1.**	7.645	Hexadecanoic acid, trimethylsilyl ester	$C_{19}H_{40}O_2Si$	328.28
**2.**	17.795	n-Octacosane	$C_{28}H_{58}$	394.45
**3.**	17.864	beta-Sitosterol trimethylsilyl ether	$C_{32}H_{58}OSi$	486.43
**4.**	19.364	Propanoic acid, 3,3'-thiobis-, didodecyl ester	$C_{30}H_{58}O_4S$	514.41

Table S2: GC-MS analysis of the total crude extract of *S. officinarum* after TMSi derivation.

PEAK	RT	COMPOUND NAME	FORMULA	MOL. MASS
**1.**	7.645	Hexadecanoic acid, trimethylsilyl ester	$C_{19}H_{40}O_2Si$	328.28
**2.**	8.840	Silane, [(3,7,11,15-tetramethyl-2-hexadecenyl)oxy]trimethyl-	$C_{23}H_{48}OSi$	368.35
**3.**	8.829	Silane, trimethyl[[5-methyl-2-(1-methylethyl)cyclohexyl]oxy]-	$C_{13}H_{28}OSi$	228.19
**4.**	9.190	11-trans-Octadecenoic acid, trimethylsilyl ester	$C_{21}H_{42}O_2Si$	354.30
**5.**	9.383	Octadecanoic acid, trimethylsilyl ester	$C_{21}H_{44}O_2Si$	356.31
**6.**	13.927	D-(+)-Trehalose, octakis(trimethylsilyl) ether	$C_{36}H_{86}O_{11}Si_8$	918.43
**7.**	14.164	Squalene	$C_{30}H_{50}$	410.39
**8.**	17.526	Ergosta-7-en-3β-ol	$C_{31}H_{56}OSi$	472.41
**9.**	17.762	Stigmasterol trimethylsilyl ether	$C_{32}H_{56}OSi$	484.41
**10.**	18.270	beta-Sitosterol trimethylsilyl ether	$C_{32}H_{58}OSi$	486.43

Table S3: GC-MS analysis of *n*-hexane fraction of *S. officinarum.*

PEAK	RT (min)	COMPOUND NAME	FORMULA	MOL. wt
**1.**	3.560	Phenol, 2,4-bis(1,1-dimethylethyl)-	$C_{14}H_{22}O$	206.17
**2.**	5.761	Tetradecanoic acid	$C_{14}H_{28}O_2$	228.21
**3.**	5.919	Bicyclo[3.1.1]heptane, 2,6,6-trimethyl-, [1R-(1.alpha.,2.beta.,5.alpha.)]-	$C_{10}H_{18}$	138.14
**4.**	6.291	3,7,11,15-Tetramethyl-2-hexadecen-1-ol	$C_{20}H_{40}O$	296.31
**5.**	7.194	n-Hexadecanoic acid	$C_{16}H_{32}O_2$	256.24
**6.**	7.713	Hexadecanoic acid, trimethylsilyl ester	$C_{19}H_{40}O_2Si$	328.28
**7.**	8.829	(*Z,Z*)-9,12-Octadecadienoic acid	$C_{18}H_{32}O_2$	280.24
**8.**	12.348	Bis(2-ethylhexyl) phthalate	$C_{24}H_{38}O_4$	390.28
**9.**	14.175	Squalene	$C_{30}H_{50}$	410.39
**10.**	16.522	dl-alpha-Tocopherol	$C_{29}H_{50}O_2$	430.38
**11.**	18.247	beta-Sitosterol	$C_{29}H_{50}O$	414.39
**12.**	18.907	D:C-Friedo-B':A'-neogammacer-9(11)-ene, 3-methoxy-, (3.beta.)-	$C_{31}H_{52}O$	440.40
**13.**	20.706	Propanoic acid, 3,3'-thiobis-, didodecyl	$C_{30}H_{58}O_4S$	514.41

Table S4: GC-MS analysis of the ethyl acetate fraction of *S. officinarum*

PEAK	RT	COMPOUND NAME	FORMULA	MOL. MASS
**1.**	19.330	Propanoic acid, 3,3'-thiobis-, didodecyl ester	$C_{30}H_{58}O_4S$	514.41

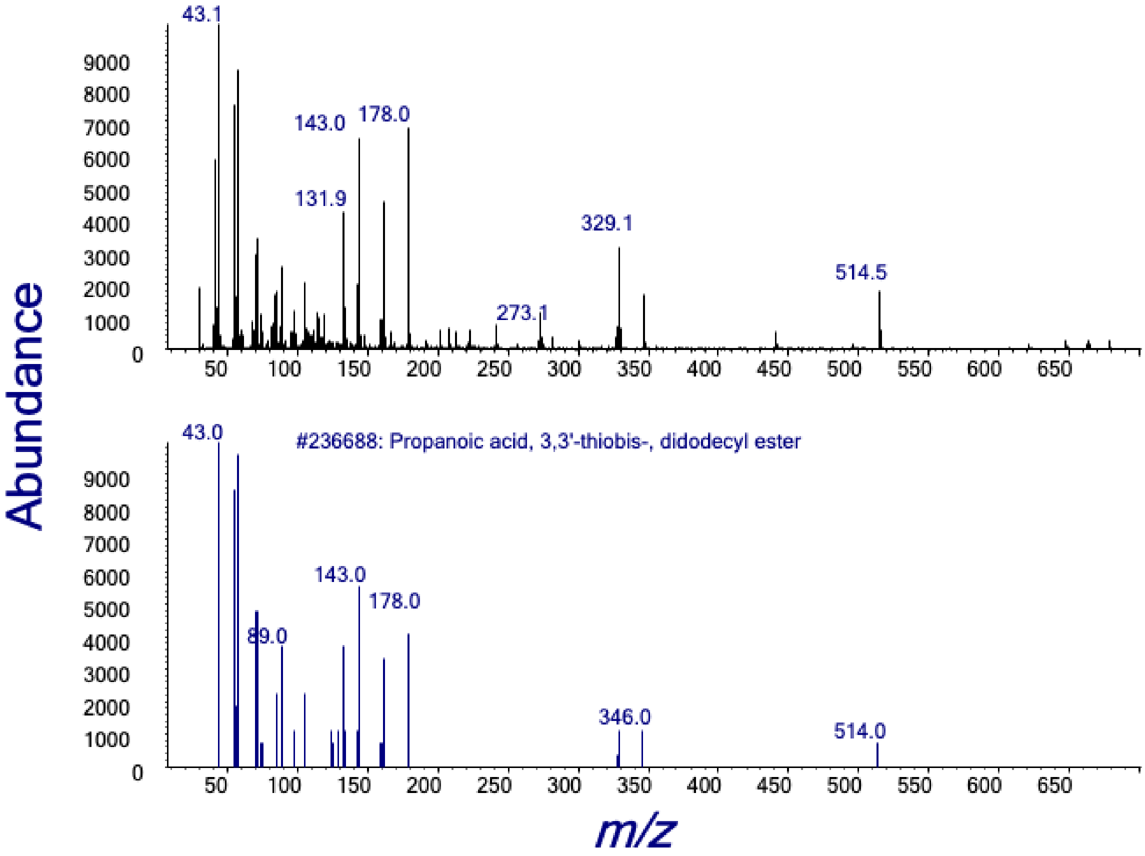

Figure S1: Comparison of GC-Mass spectrum of dilauryl thiodipropionate (**4**) (top) from DCM fraction of *S. officinarum* with that of dilauryl thiodipropionate in NIST library (bottom).

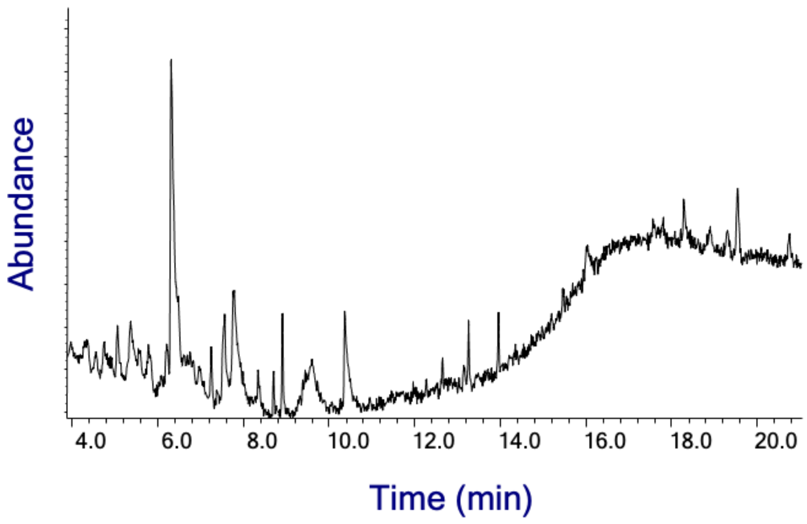

Figure S2: GC-MS chromatogram of *n*-butanol fraction of *Saccharum offinarium* (SO) after TMSi derivation.

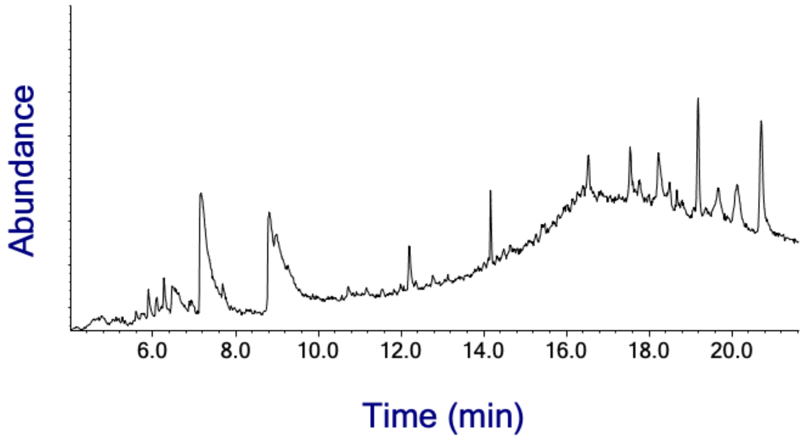

Figure S3: GC-MS chromatogram of *n*-hexane fraction of *Saccharum offinarium.*

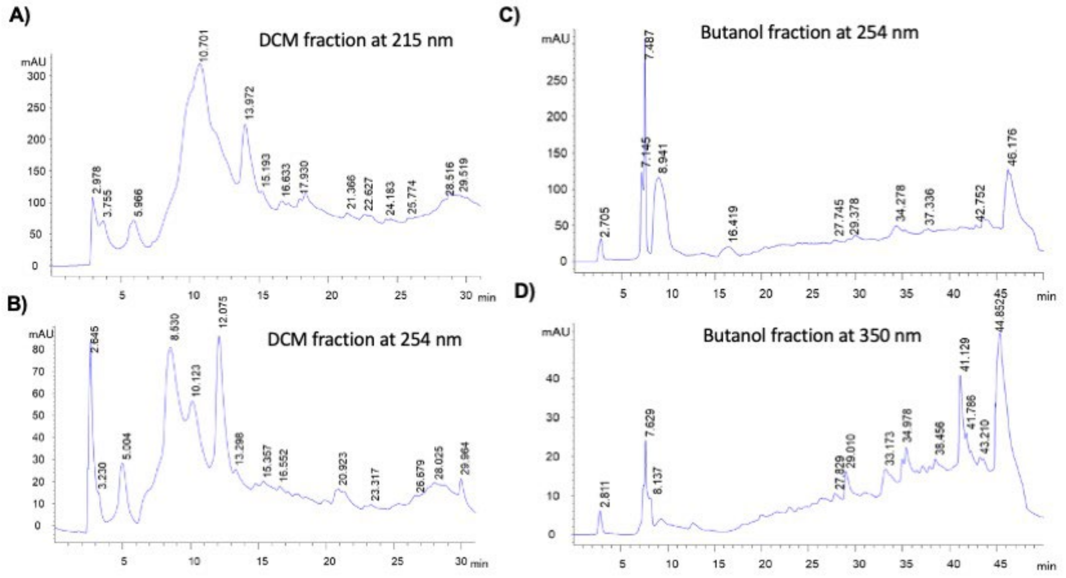

Figure S4: Analytical HPLC profile of the DCM fraction of Sugarcane leave extracts at wavelength of 215 nm (A), 254 nm (B); and the butanol fraction at 254 nm (C) and 350 nm (D).

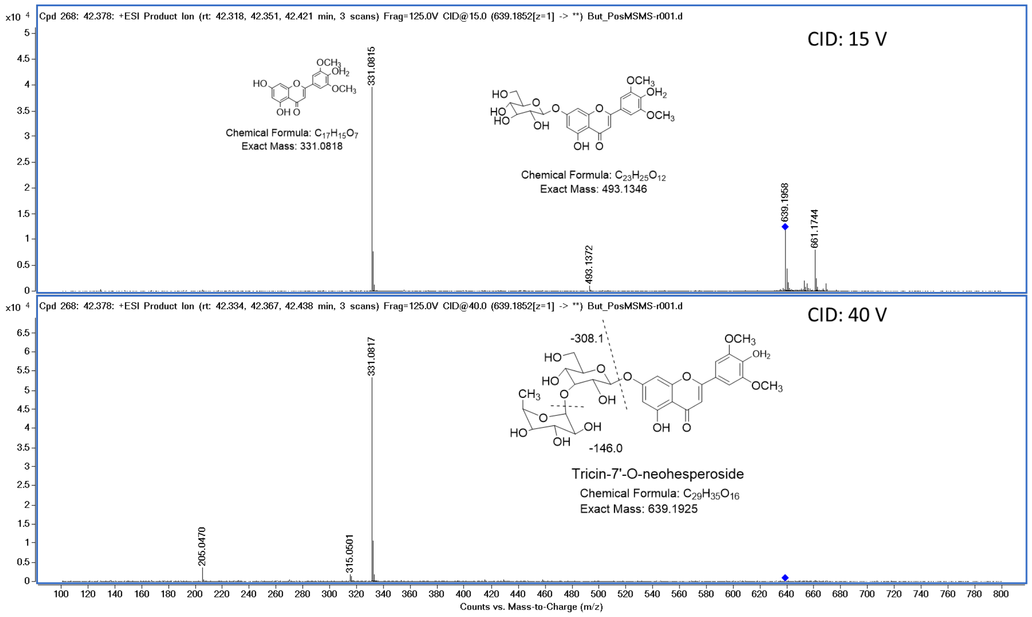

Figure S5. LC-TOF-(positive mode) MS/MS spectrum of tricin-7-O-eohesperoside from the butanol fraction of sugarcane leave extracts. CID-MS/MS spectra (15V, top; 40 V bottom) of mass 639.1852 $[M+H]^+$ indicating a major common fragment ion at *m/z* 331.0817 through the loss of rhamnose and glucose, and a minor ion at *m/z* 493.1372 through the loss of rhamnose (-146) at 15 V.

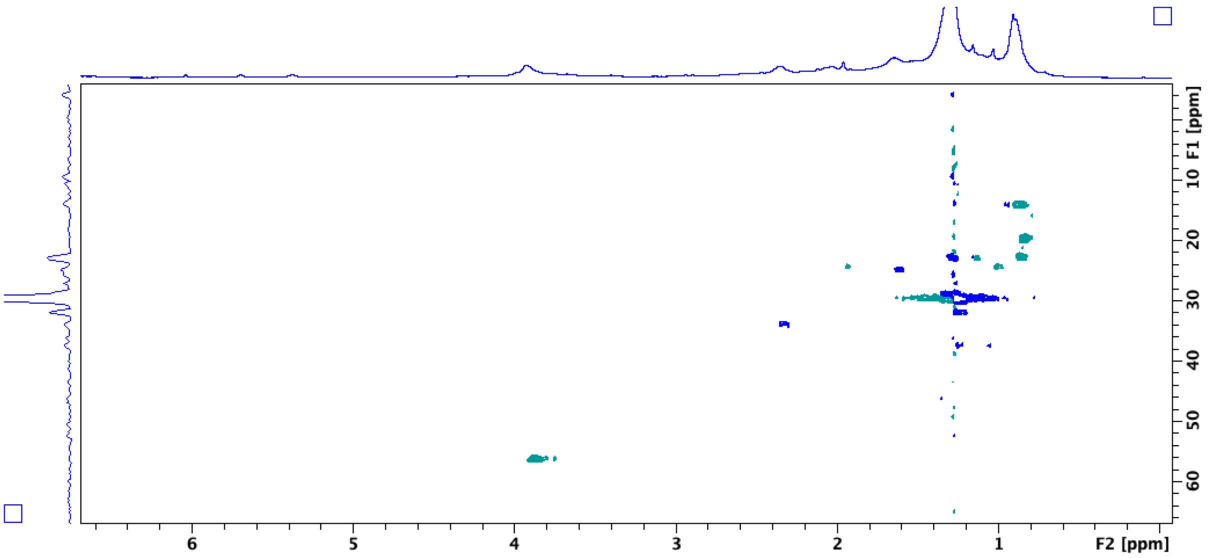

Figure S6. HSQC of DCM fraction ($CDCl_3$) indicating the presence of long chain of fatty acids and methoxy groups.

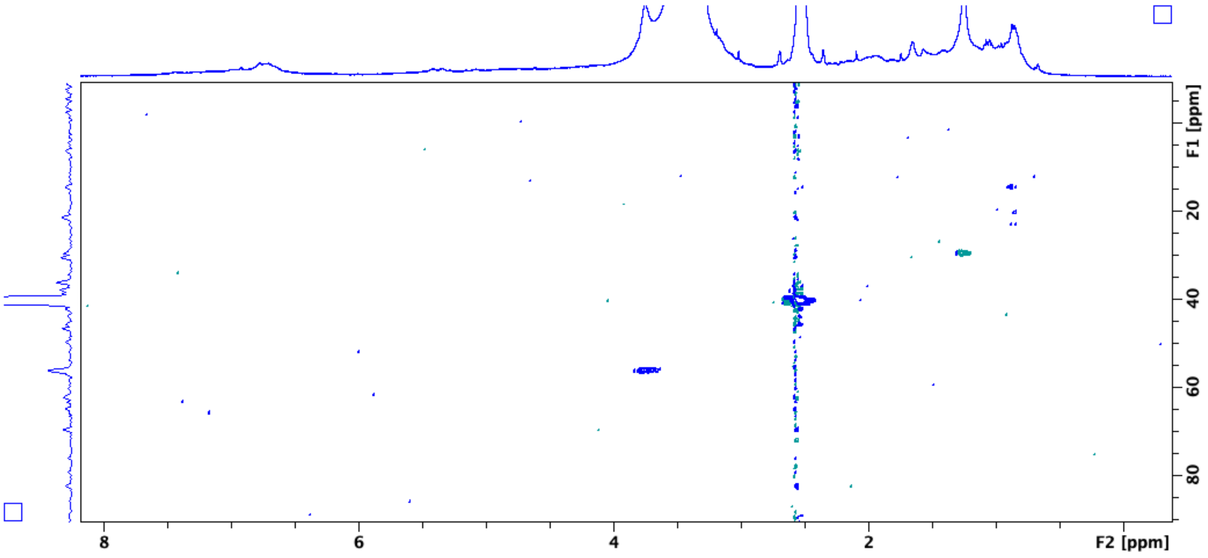

Figure S7. HSQC of the butanol fraction (DMSO-d_6) indicating the presence of methoxy groups likely from the flavonoids such as tricin-7-O-neohesperidoside and 3,4',5,6,7-pentamethoxyflavone.
